# CZT-1 Is a Novel Transcription Factor Controlling Cell Death and Natural Drug Resistance in *Neurospora crassa*

**DOI:** 10.1534/g3.114.011312

**Published:** 2014-04-08

**Authors:** A. Pedro Gonçalves, Charles Hall, David J. Kowbel, N. Louise Glass, Arnaldo Videira

**Affiliations:** * IBMC, Instituto de Biologia Molecular e Celular, Universidade do Porto, Rua do Campo Alegre 823, 4150-180 Porto, Portugal; † ICBAS, Instituto de Ciências Biomédicas de Abel Salazar, Universidade do Porto, Rua de Jorge Viterbo Ferreira 228, 4050-313 Porto, Portugal; ‡ Plant and Microbial Biology Department, The University of California, Berkeley, 341 Koshland Hall, 94720, Berkeley, California

**Keywords:** cell death, multidrug resistance, zinc cluster transcription factor, genome-wide association study, RNA-seq

## Abstract

We pinpoint CZT-1 (cell death–activated zinc cluster transcription factor) as a novel transcription factor involved in tolerance to cell death induced by the protein kinase inhibitor staurosporine in *Neurospora crassa*. Transcriptional profiling of staurosporine-treated wild-type cells by RNA-sequencing showed that genes encoding the machinery for protein synthesis are enriched among the genes repressed by the drug. Functional category enrichment analyses also show that genes encoding components of the mitochondrial respiratory chain are downregulated by staurosporine, whereas genes involved in endoplasmic reticulum activities are upregulated. In contrast, a staurosporine-treated Δ*czt-1* deletion strain is unable to repress the genes for the respiratory chain and to induce the genes related to the endoplasmic reticulum, indicating a role for CZT-1 in the regulation of activity of these organelles. The Δ*czt-1* mutant strain displays increased reactive oxygen species accumulation on insult with staurosporine. A genome-wide association study of a wild population of *N. crassa* isolates pointed out genes associated with a cell death role of CZT-1, including catalase-1 (*cat-1*) and apoptosis-inducing factor–homologous mitochondrion-associated inducer of death 2 (*amid-2*). Importantly, differences in the expression of *czt-1* correlates with resistance to staurosporine among wild isolate strains. Our results reveal a novel transcription factor that regulates drug resistance and cell death in response to staurosporine in laboratory strains as well as in wild isolates of *N. crassa*.

In 1958, Strauss observed that certain auxotrophic mutant strains of *Neurospora crassa* committed “suicide” when a required growth factor was absent from the culture medium (Strauss 1958). This suicide response was only true for mutant strains that tried to initiate growth by synthesizing protein and nucleic acids despite the lack of the compound that they required to grow. Strains that did not attempt to initiate growth did not die; therefore, it was stated that cell death was due to unbalanced growth. To our knowledge, this was the first description of the occurrence of cell death in *N. crassa*. More recently, *in silico* analysis showed that when compared to typical models of cell death such as yeasts, filamentous fungi possess additional homologs of mammalian mediators of cell death. The degree of homology to executioner proteins of mammalian cell death is also higher in filamentous fungi, suggesting that these organisms are an attractive alternative option to study this biological process (Fedorova *et al.* 2005). Research involving *N. crassa* is well-supported by a large set of tools and techniques, namely the collection of deletion mutants (Colot *et al.* 2006; Dunlap *et al.* 2007) and its long history as a classical model organism for cell biology and genetics.

In *N. crassa*, cell death can be triggered genetically through the fusion of hyphae or germlings of two incompatible individuals at *het* loci (Hutchison *et al.* 2009, 2012) or induced with phytosphingosine (Castro *et al.* 2008; Fernandes *et al.* 2013; Videira *et al.* 2009), staurosporine (Castro *et al.* 2010; Fernandes *et al.* 2011, 2013), hydrogen peroxide (Castro *et al.* 2008), chitosan (Palma-Guerrero *et al.* 2009), or PAF26 (Munoz *et al.* 2012). It can also be stimulated through ectopic expression of the *phcA* gene from *Pseudomonas syringae* (Wichmann *et al.* 2008) or by a combined stimulus of heat shock (45°) with 2-deoxyglucose-induced glucose deprivation (Plesofsky *et al.* 2008; Plesofsky-Vig and Brambl 1995). In particular, we observed that staurosporine induces a cell death program that includes several cellular alterations such as loss of viability, chromatin fragmentation, early reactive oxygen species (ROS) production, and glutathione (GSH) export (Castro *et al.* 2010; Fernandes *et al.* 2013; Gonçalves and Videira 2014). The combined treatment with staurosporine and the classical mitochondrial complex I inhibitor rotenone (which is also an anti-mitotic agent) resulted in a synergistic inhibitory effect in the growth of *Neurospora crassa*, *Aspergillus fumigatus*, *Candida albicans* (Castro *et al.* 2010), and human thyroid cancer cells (Gonçalves *et al.* 2011a,b). Thus, this filamentous fungus has great potential for modeling programmed cell death and for investigating new strategies of therapy in clinically relevant contexts. The mediators of cell death in *N. crassa*, both regulators and executioners, are still largely unknown.

Zinc cluster transcription factors are important fungal regulators of diverse cellular functions such as sugar and amino acid metabolism, filamentous growth, chromatin remodeling, carbon source response, ergosterol biosynthesis, and multidrug resistance (MacPherson *et al.* 2006). Here, we show that a novel transcription factor encoded by NCU09974 and termed CZT-1 (for cell death–activated zinc cluster transcription factor) is a major regulator of cell death in *N. crassa*. It controls the expression of several genes whose protein products affect different organelles, such as the mitochondria and the endoplasmic reticulum, as well as several functions, including ROS accumulation. Through its transcriptional regulation, CZT-1 confers resistance to staurosporine-induced cell death in laboratory strains and in wild isolates of *N. crassa*.

## Materials and Methods

### Strains, culture media, and chemicals

Standard procedures for the handling of *N. crassa* cells were used. Cells were grown in Vogel’s minimal medium plus 1.5% (w/v) sucrose (Vogel 1956). Agar at 1.5% (w/v) concentration was added to obtain solid medium. Wild-type (including natural isolates) and deletion strains are available from the Fungal Genetics Stock Center (McCluskey *et al.* 2010). All 112 strains used for the genome-wide association study were isolated from Louisiana (USA) and described by Palma-Guerrero *et al.* (2013). The following chemicals were used: staurosporine (LC Laboratories); dimethyl sulfoxide (DMSO); hydrogen peroxide; cinnamic acid; amphotericin B (Sigma-Aldrich); and phytosphingosine (Avanti Polar Lipids).

### Growth assays

Hyphal growth at 26° was obtained by measuring colony elongation over time after the inoculation of 20 μl with 5×10^4^ conidia in the center of a large Petri dish (14.2 cm diameter) containing solid minimal medium. For growth assessment in liquid minimal medium, 1×10^4^ conidia/ml were incubated at 26°, at 100 rpm, under constant light in 96-well plates (200 μl/well). Absorbance at 620 nm was followed during 24 hr and the percentage of growth for each condition *vs.* the control was calculated. For the spot assay, nine serial three-fold dilutions were prepared for each strain starting with 6.6×10^7^ cells/ml, so that the last spot contained approximately 50 conidia; 5 μl from each dilution was spotted separately on plates containing glucose-fructose-sorbose medium with agar (GFS) supplemented with the appropriate chemicals. Cells were incubated at 26° and pictures were taken ∼72 hr after inoculation.

### Semi-quantitative real-time PCR

Conidia at a concentration of 1×10^6^ cells/ml were grown in minimal medium for 5 hr at 30°, followed by the addition of staurosporine or DMSO, and then grown for an additional hour. Cells were harvested by centrifugation (5000 rpm, 5 min) and RNA isolation was performed using a PureLink RNA Mini kit (Life Technologies) or a ZR Fungal/Bacterial RNA MicroPrep kit (Zymo Research) according to the manufacturer’s protocol; 1 μg of RNA was used for cDNA preparation using the SuperScript First-Strand Synthesis System kit (Life Technologies) following the provided protocol. A mix containing SYBR Green (Bio-Rad) and specific primers (*czt-1* FW: GGTGACAACGACGACGAGATGG; *czt-1* RV: GAGTCCTGGTTAGTTCGCTTACGG; *actin* FW: GGCATCACACCTTCTACAACGAG; *actin* RV: ATGTCAACACGGGCAATGGC) was prepared and amplification was performed with a Bio-Rad iCycler iQ5. Triplicates were obtained in each experiment and threshold cycle values within an interval of ±0.5 cycles in the same experiment were accepted. Relative gene expression was determined by the 2^−ΔΔCt^ method (Livak and Schmittgen 2001). For the quantifications, *actin* (NCU04173) was used as control and cDNA from the wild-type strain not exposed to staurosporine was used as calibrator. For the time-course analysis of the basal expression of *czt-1* during germination, no calibrator was used and gene expression was calculated using 2^−ΔCt^.

### Reactive oxygen species determination

The production of ROS was measured using the fluorescent probe dihydrorhodamine 123 (Sigma-Aldrich). After growing 10^6^ conidia/ml for 4 hr in minimal medium at 26°, 20 μg/ml dihydrorhodamine 123 and staurosporine (or DMSO) were added for another 30 min. Samples were harvested by centrifugation and washed twice with PBS before being resuspended in PBS and read in a FACS Calibur (BD) flow cytometer. The resulting files were analyzed with FlowJo (Tree Star).

### Statistical analysis

Statistical analysis of the data was performed using SPSS 20 (SPSS Inc.). The non-parametric Mann-Whitney test was used for comparisons between two groups unless otherwise stated. P-values ≤0.05 were considered statistically significant.

### High-throughput RNA sequencing

Conidial suspensions at 1×10^6^ cells/ml were incubated in minimal medium for 6 hr (26°, 140 rpm, constant light) followed by the addition of staurosporine (or DMSO) for an additional hour. Cells were harvested using 0.45-μm filters and immediately frozen in liquid nitrogen. Total RNA was isolated by the Trizol-Phenol-Chloroform method. After digestion of 25 μg RNA with TURBO DNAse (Life Technologies), mRNA was purified using Dynabeads oligo(dT) magnetic beads (Life Technologies). The mRNA was chemically fragmented using the Ambion RNA fragmentation kit (Life Technologies). First and second strand cDNA synthesis was achieved using appropriate kits (Life Technologies). The illumina TruSeq kit was used to generate the cDNA libraries with indexing adapters following the manufacturer’s protocol. After purification of the libraries with AMPure XP beads (Roche), the quality of the libraries was analyzed in an Agilent 2100 Bioanalyzer. The cDNA libraries were sequenced in an Illumina HiSeq2000 and single reads of 50 bp were obtained. Sequencing data were handled with Tophat, Cufflinks, and Cuffdiff (Trapnell *et al.* 2012). Expression levels are presented as fragments/reads per kilobase of transcript per million mapped reads (FPKM/RPKM). The resulting dataset is available at the NCBI GEO database (http://www.ncbi.nlm.nih.gov/geo/; series record: GSE52153). CummeRbund (Trapnell *et al.* 2012) was used to generate scatter plots and volcano plots. Functional enrichment of sets of genes was assessed with FunCat (Ruepp *et al.* 2004) or BLAST2GO (Conesa *et al.* 2005).

### Single nucleotide polymorphism identification

Maq (Li *et al.* 2008) was used to map RNA sequencing (RNA-seq) reads to the genome sequence of *N. crassa* strain FGSC 2489 (Galagan *et al.* 2003) and for the identification of single nucleotide polymorphisms (SNPs). RNA-seq reads that mapped to multiple locations or SNPs located in regions of low consensus read quality were eliminated from analysis. Only those that were bi-allelic were retained, yielding a complete data set of 1.09×10^6^ SNPs. The complete SNP set used in this analysis was previously published (Palma-Guerrero *et al.* 2013). For markers used as input into calculations of genetic association with the staurosporine resistance phenotype, we filtered the complete SNP set to retain only sites at which the minor allele was present at >10% frequency.

### Genome-wide association study

The expression level of CZT-1 from the Louisiana population of *N. crassa* wild strains (Ellison *et al.* 2011; Palma-Guerrero *et al.* 2013) was used as a quantitative proxy for staurosporine resistance (please see *Results* for details). For each strain, the normalized expression level of *czt-1* (as RPKMs) was compared to the mean for all strains. A strain showing higher than average expression of *czt-1* (46 strains) was considered to have “high” expression of *czt-1* and was binned as more resistant to staurosporine. Conversely, a strain showing lower than average expression of *czt-1* (67 strains) was considered to have “low” expression of *czt-1* and was binned as less resistant to staurosporine. Each SNP marker was then tested, in turn, from our set of SNPs with >10% minor allele frequency, for co-inheritance with qualitative determination of “high” score using Fisher exact test. Significance values were determined using an empirical null distribution of P-values from the observed data. For this, we performed 1000 permutations. For each permutation, the phenotype category was shuffled relative to the genotypes and the minimum P-value was retained. Any nominal P-value larger than a minimum observed permuted P-value was considered as a possible false positive and removed from consideration.

### Protein sequence analysis

The following bioinformatics tools were used for alignments, domain prediction, and phylogenetic tree building: NCBI BLAST (Altschul *et al.* 1990); ClustalW2 (Larkin *et al.* 2007); InterProScan (Quevillon *et al.* 2005); MEMSAT (Jones 2007); TMHMM (Krogh *et al.* 2001); and Mega5 (Tamura *et al.* 2011).

## Results

### NCU09974/*czt-1* is involved in resistance to staurosporine and reactive oxygen species accumulation

Staurosporine induces cell death in *N. crassa* accompanied by typical features like loss of viability, DNA fragmentation, ROS production, and GSH export (Castro *et al.* 2010; Fernandes *et al.* 2013). Previously, by screening strains from the *N. crassa* deletion collection (Colot *et al.* 2006) for sensitivity to staurosporine, we observed that a strain lacking NCU09974 was highly susceptible to the drug (Fernandes *et al.* 2011). Functional NCU09974 seems to influence the transcription of at least two genes (Fernandes *et al.* 2011), although this protein was not previously characterized or considered in transcription factor–related works in Neurospora.

**Figure 1:**
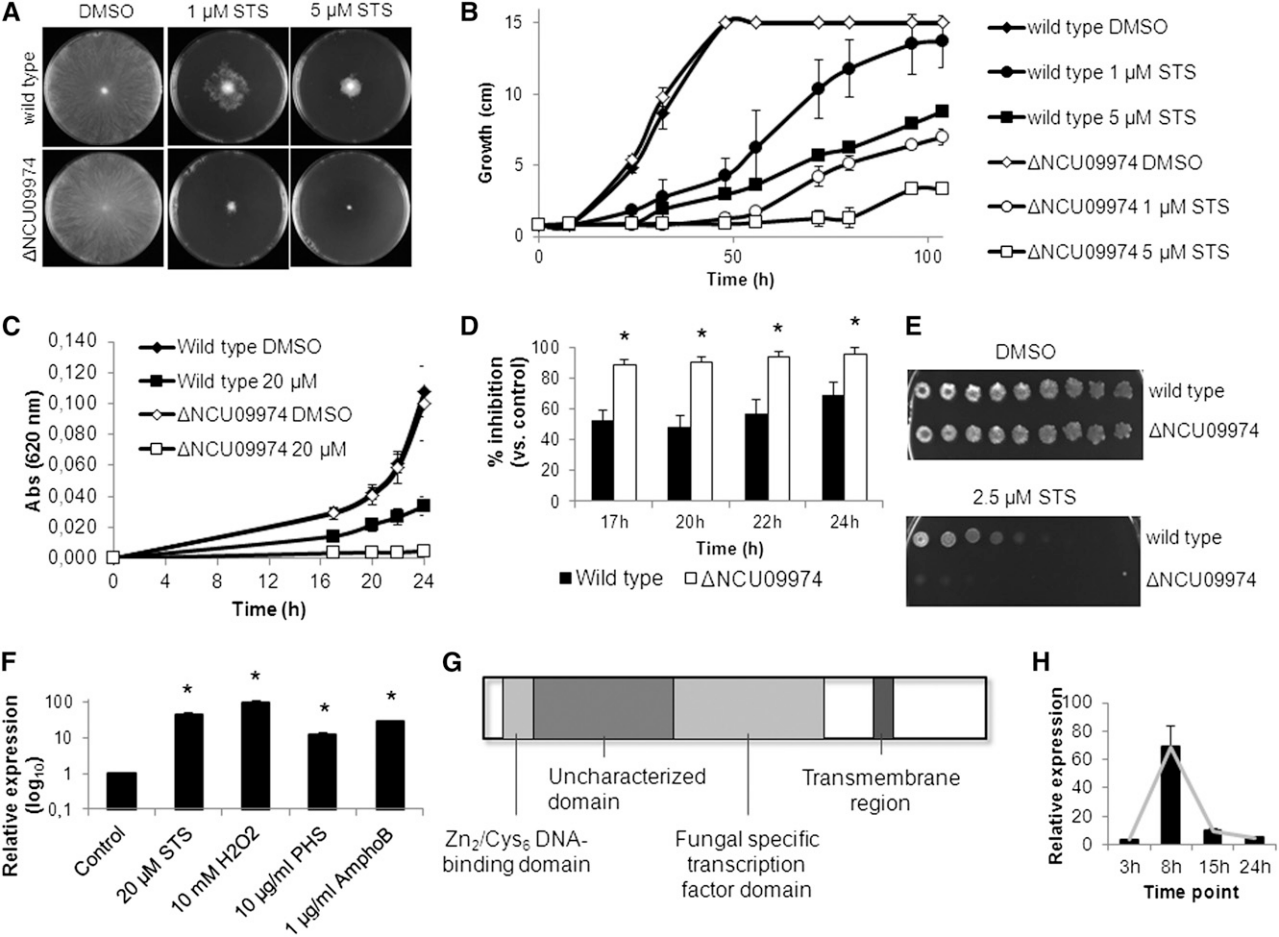
Deletion of NCU09974/*czt-1* confers sensitivity to staurosporine. (A and B) Conidia from the wild-type and ΔNCU09974 strains were inoculated in the center of Petri dishes containing solid minimal medium supplemented with staurosporine (STS) or DMSO. Radial growth at 48 hr (A) and radial growth throughout time (B) are shown. (C and D) Growth of the strains on liquid minimal medium followed by measuring absorbance at 620 nm (C) and percentage of growth inhibition caused by STS (D). *P-value <0.05. (E) Sensitivity of the strains to STS was tested by spotting conidia in GFS medium. (F) Relative expression of NCU09974 was quantified by qRT-PCR after exposing wild-type cells to STS, hydrogen peroxide (H_2_O_2_), phytosphingosine (PHS), and amphotericin B (AmphoB) for 1 hr. (G) Diagram showing the main predicted sequence features of CZT-1. (H) Time-course relative expression of *czt-1* as quantified by qRT-PCR during germination of wild-type conidia at 26°.

We confirmed that lack of NCU09974 results in hypersensitivity to staurosporine-induced cell death. We inoculated the wild-type and the deletion strain for NCU09974 (ΔNCU09974) on solid Vogel’s minimal medium supplemented with staurosporine and observed that the latter is much more affected by the drug (Figure 1, A and B). The same sensitivity to staurosporine is observed when ΔNCU09974 is grown in liquid Vogel’s minimal medium (Figure 1, C and D) or spotted on GFS plates (Figure 1E), indicating that NCU09974 is a key player in resistance to staurosporine-induced cell death.

Our previous microarray data indicated that expression of NCU09974 is stimulated by exposure to staurosporine (Fernandes *et al.* 2011) and phytosphingosine (Videira *et al.* 2009). Quantitative RT-PCR experiments show that the gene is upregulated not only by staurosporine and phytosphingosine but also by hydrogen peroxide treatment after oxidative stress insult or by the antifungal amphotericin B (Figure 1F). Furthermore, expression of NCU09974 is also induced on exposure to menadione (Zhu *et al.* 2013) and by two novel cell death–inducing compounds currently undergoing characterization in our group (data not shown).

The sensitivity of ΔNCU09974 to several other chemical compounds was tested with the spot assay. Inhibition of growth of ΔNCU09974 is similar to wild-type when the strains are treated with actinomycin D, hydrogen peroxide, deoxycholic acid, acetic acid, ethanol, paraquat, imidazole, cycloheximide, dithiothreitol, 8-hydroxyquinoline, diphenyleneiodonium, menadione, antimycin A, oligomycin, or caffeine (data not shown). However, ΔNCU09974 is slightly more sensitive than wild-type to phytosphingosine and cinnamic acid (Supporting Information, Figure S1, A and B) and more resistant to amphotericin B (Figure S1C). These data indicate that NCU09974 is activated by cell death stimuli and its absence is especially crucial for the response to staurosporine, likely because of specific pathways activated by this drug.

Conserved domain prediction on the protein sequence of CZT-1 shows the presence of a Zn_2_/Cys_6_ DNA-binding domain, characteristic of zinc cluster transcription factors, near the N-terminal part of the protein and a “fungal-specific transcription factor domain” in the middle of the molecule (Figure 1G). The function of this fungal-specific “transcription factor domain” in this family of proteins is unclear. Between these two domains, an “uncharacterized transcriptional regulatory protein” domain is found. A transmembrane segment is also robustly predicted by different bioinformatics tools. Because NCU09974 displays typical features of zinc cluster transcription factors and because our results implicate it as a mediator of the fungal response to cell death, we named it CZT-1 (cell death–activated zinc cluster transcription factor).

The time-course expression of *czt-1* during *N. crassa* germination in liquid Vogel’s minimal medium is shown in Figure 1H. It reveals a maximum level of expression after 8 hr in accordance with microarray data that included *czt-1* in the list of differentially expressed genes during asexual development of Neurospora (Greenwald *et al.* 2010). However, parameters such as aerial hyphae elongation, conidial production, growth rate, and germination are not affected by the deletion of *czt-1* (data not shown).

**Figure 2:**
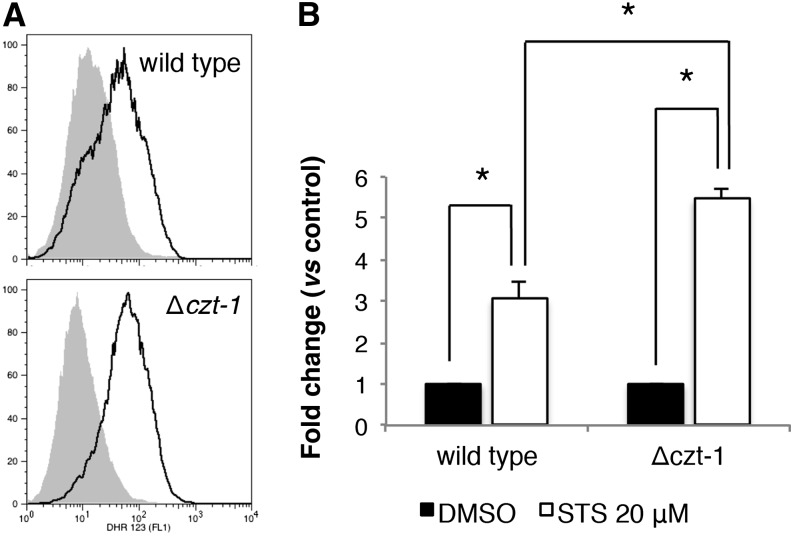
CZT-1 is involved in ROS accumulation. (A and B) ROS accumulation was assessed with dihydrorhodamine 123 by staining cells grown in liquid minimal medium for 4 hr and treated with staurosporine (STS) or DMSO for 30 min. Representative flow cytometry histograms are shown in (A) (solid line, STS; shaded, DMSO) and the respective quantification is shown in (B). *P-value <0.05.

Staurosporine-induced cell death in *N. crassa* is ROS-dependent because addition of the antioxidant GSH results in suppression of cell death (Castro *et al.* 2010). We observed that the Δ*czt-1* mutant accumulates more ROS than wild-type on insult with staurosporine. ROS accumulation increases approximately three times in the wild-type and 5.5-times in the knockout mutant (Figure 2, A and B). This function of CZT-1 in the control of ROS accumulation is in agreement with its induction by hydrogen peroxide (Figure 1G). These data suggest a function for CZT-1 in the control of ROS accumulation, although it is unclear if it acts at the level of ROS production or detoxification.

### Natural variation to drug resistance in *N. crassa*

Two natural and divergent populations of *N. crassa* from the region of the Caribbean basin were recently described based on SNP search after whole-transcriptome sequencing (Ellison *et al.* 2011). We examined the expression levels of *czt-1* in 111 strains from one of these populations (the subtropical Louisiana population) and also of the wild-type laboratory strain (FGSC 2489). We observed minimum, maximum, and median RPKM of 10.14, 125.06, and 29.19, respectively (Figure S2), demonstrating that there is variation in the expression of this gene within a single population of wild isolates.

**Figure 3:**
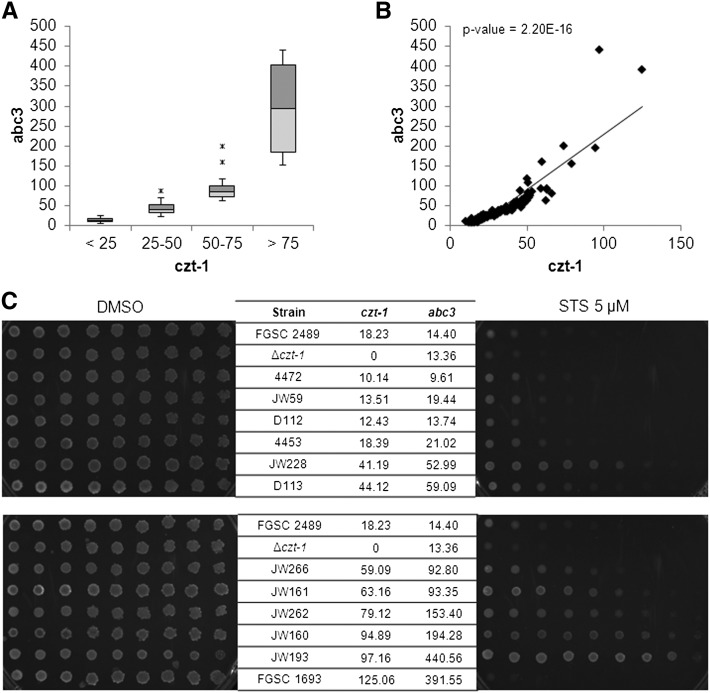
CZT-1 controls resistance to cell death in wild isolates of *N. crassa*. (A) Expression levels of *abc3* in wild strains. The strains were separated in four groups based on *czt-1* expression (<25; 25–50; 50–75, and >75 *czt-1* RPKM). (B) Correlation between the expression levels of *czt-1* and *abc3* in a scatter plot. (C) Staurosporine (STS) sensitivity of wild strains representing each of the four groups presented in (B) was tested by spot assays on GFS media supplemented with the drug. The laboratory strain FGSC 2489 was used as a reference. The expression levels of *czt-1* and *abc3* in each strain are indicated.

We recently observed that CZT-1 influences the expression of NCU09975, which encodes the multidrug resistance–related pump ABC3 (Fernandes *et al.* 2011). Activation of CZT-1 may lead to the upregulation of *abc3*, so we hypothesized that wild isolates of the Louisiana population with higher levels of *czt-1* would also have higher expression of *abc3*. We divided the strains in four groups based on the RPKM values for *czt-1* (<25, 25–50, 50–75, and >75) and considered the respective RPKM for *abc3*. There is a clear correlation between the expression of these two genes (Figure 3, A and B), arguing in favor of a regulatory role of CZT-1 on *abc3*. Because the lack of *czt-1* or *abc3* leads to hypersensitivity to staurosporine (Fernandes *et al.* 2011), we hypothesized that, conversely, overexpression of both would lead to drug resistance. This would also support the conclusion that the hypersensitivity of Δ*czt-1* cells (Figure 1, A–E) is due to the lack of CZT-1. To test this, we selected some strains representing the four groups of *czt-1* RPKMs and assessed their sensitivity to staurosporine. Wild isolates with higher levels of expression of *czt-1* and *abc3* show increased resistance to the drug (Figure 3C), arguing in favor of a specific role of CZT-1. The same strains also show different sensitivity to oxidative stress with hydrogen peroxide (data not shown). FGSC 1693 is an exception, presumably due to alterations in other genes with a role in tolerance to staurosporine. These data indicate that there is natural variation in drug resistance among wild isolates of Neurospora.

**Figure 4:**
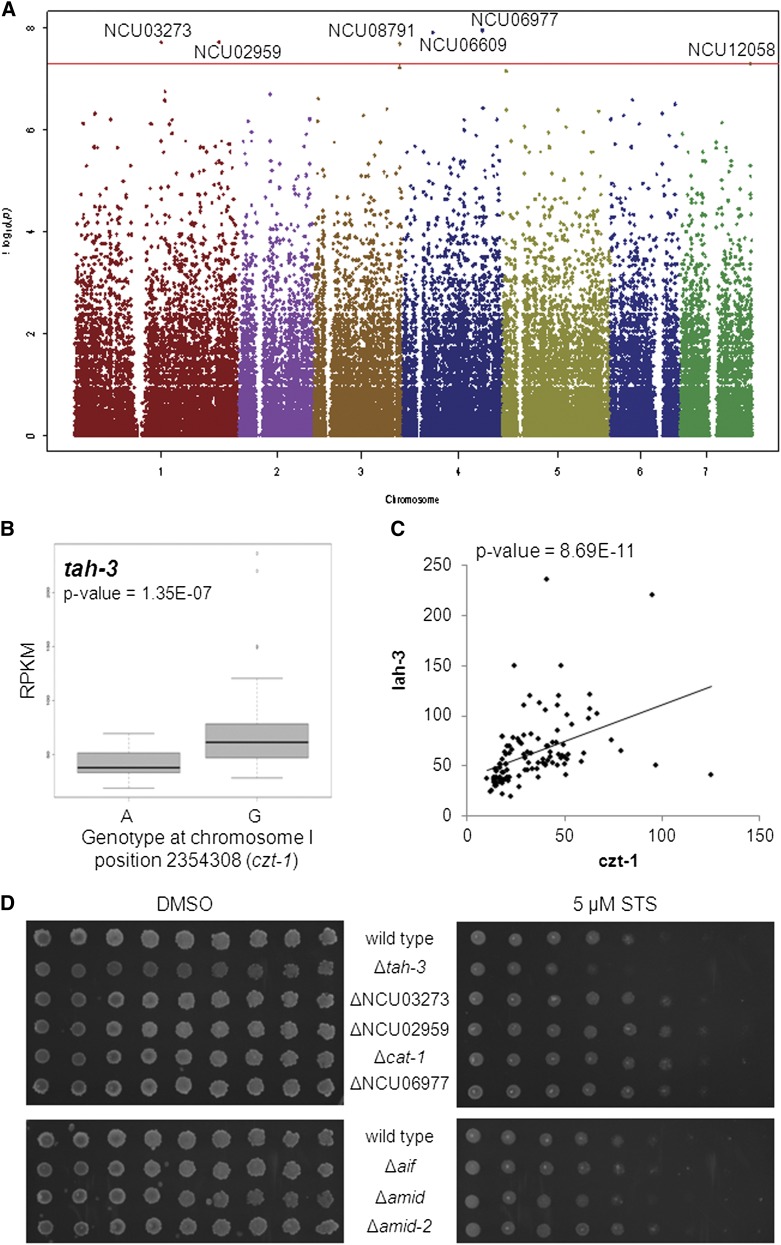
Genome-wide association studies (GWAS) links CZT-1 to novel putative mediators of cell death in Neurospora. (A) Manhattan plot depicting the results of a GWAS between the expression of *czt-1* and the presence of SNPs in the Louisiana population of wild isolates. The dots are the -log of the P-value from the Fisher’s exact test for each SNP. Because the strongest associations have the smallest P-values, their negative logarithms are the greatest. The horizontal axis represents the seven chromosomes of *N. crassa*. The red line is the significance cut-off as determined by permutation tests. (B) A second GWAS found an association between a SNP (A/G) in *czt-1* and the expression of *tah-3*. (C) Correlation between the expression levels of *czt-1* and *tah-3* in a scatter plot. (D) The spot assay on GFS plates supplemented with STS was used to test the sensitivity of deletion strains for the genes identified by the GWAS.

Genome-wide association studies (GWAS) allow the analysis of phenotypic traits whose variation is determined by genotypic differences (McCarthy *et al.* 2008). GWAS normally associate traits to quantitative loci, based on SNP analyses. Given the correlation between expression of *czt-1* and resistance to staurosporine, the former was used as a quantitative proxy for drug resistance in a GWAS using the cDNA-sequenced Louisiana population of wild isolates. Six genes with SNPs significantly associated with the expression of *czt-1* were identified (Figure 4A and Table 1). Of them, NCU08791 and NCU12058 encode CAT-1 and AMID-2, which are known to be involved in the detoxification of ROS during oxidative stress (Chary and Natvig 1989) and in the execution of mammalian cell death (Wu *et al.* 2002), respectively. A more detailed analysis of all genes showed that the genotype at each SNP locus is significantly associated with altered expression of the respective gene (Table 1 and Figure S3).

**Table 1:** Genes with a single nucleotide polymorphism associated with increased expression of *czt-1* in the Louisiana population of wild isolates

**Gene**	**Gene Name**	**Annotation/Domains**	**SNP**			**Genotype Associated with Higher Expression; P-value*****^a^***
			**Linkage Group**	**Locus**	**Genotype**	
NCU03273	—	NF-X1 finger transcription factor; contains a R3H domain	I	5150086	A, G	G; 1.16E-04
NCU02959	—	Homolog to RNA-binding proteins from other fungi	I	8577711	C, T	C; 2.59E-07
NCU08791	*cat-1*	Catalase; antioxidant system	III	5113141	C, T	C; 3.44E-07
NCU06609	—	Contains a R3H domain	IV	1796142	A, G	G; 0.006564
NCU06977	—	Homology with a subunit of the F0 portion of the mitochondrial ATP synthase	IV	4746463	A, G	G; 2.66E-06
NCU12058	*amid-2*	Apoptosis-inducing factor-homologous mitochondrion-associated inducerof death 2	VII	4179144	A, G	G; 2.69E-04

a For all SNPs, a specific allele at the indicated position is associated with increased expression of the respective gene.

We also performed the reverse GWAS, *i.e.*, we looked at expression of genes linked to SNPs cis to *czt-1*. We identified a SNP (A/G) in *czt-1* that was significantly linked to the expression of NCU03686/*tah-3* (Figure 4B). The expression of *tah-3* (also designated *lah-3*) correlates well with the expression of *czt-1* (Figure 4C). TAH-3 is also a Zn_2_/Cys_6_ binuclear cluster transcription factor as *czt-1* and has been reported to be required for fungal tolerance to a harsh environment caused by an argon plasma jet (Park *et al.* 2012).

The results of both GWAS pointed out genes that, because of their genetic association with *czt-1*, may underlie the natural resistance phenotype observed for the wild strains. Therefore, we next tested the sensitivity profile of the knockout strains for the genes identified by the GWAS (except for ΔNCU06609, as the strain was unavailable). Despite the fact that it grows slightly worse than wild-type in control medium, the Δ*tah-3* deletion mutant is more sensitive to staurosporine than wild-type. ΔNCU03273, ΔNCU02959, ΔNCU06977, and Δ*cat-1* are slightly more resistant than wild-type (Figure 4D, upper panel). This further implicates these molecules in the cell death response to staurosporine and in the resistance conferred by CZT-1, although we do not currently know why the deletion strains for the genes identified by the GWAS have an opposite phenotype in response to staurosporine. The deletion strain for *amid-2* is not particularly sensitive or resistant to the drug (Figure 4D, lower panel), probably due to redundant functions of proteins of the same family, like amid and AIF (Carneiro *et al.* 2012).

Altogether, our data point to the existence of natural variation to drug resistance in wild isolates of *N. crassa* and to the relevance of the CZT-1 transcriptional regulator. The genes that interact genetically with CZT-1, indicated by the GWAS, likely contribute to the resistance phenotype of the strains.

### Transcriptional profiling of staurosporine-treated wild-type *vs.* Δ*czt-1*

**Figure 5:**
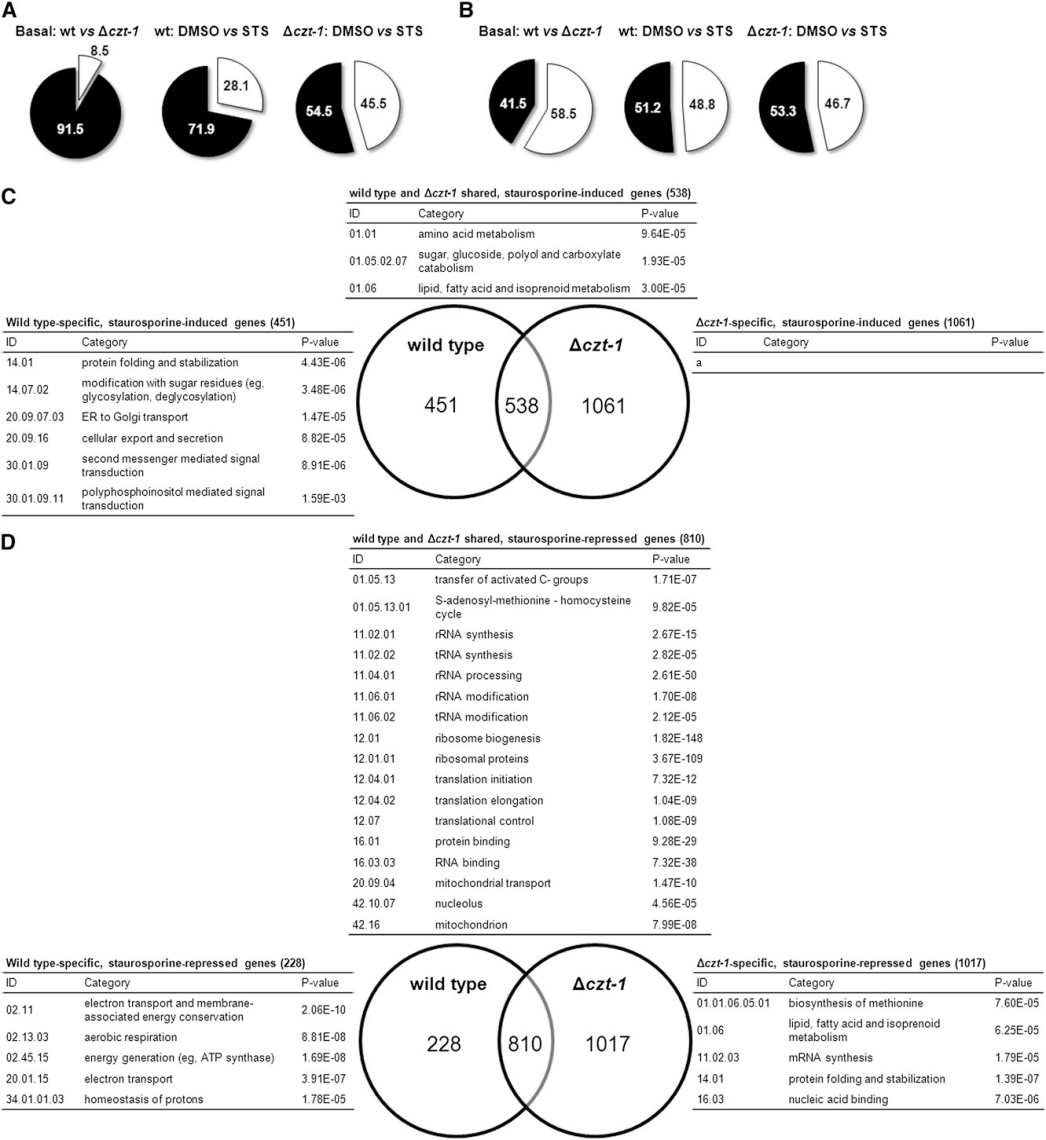
Summary of the transcriptional response to staurosporine in wild-type and Δ*czt-1* cells and functional enrichment analysis of the genes with altered expression. (A) The percentage of genes with altered expression is represented by the white portion of the pie chart. The left panel represents the basal gene expression altered in the Δ*czt-1* mutant *vs.* wild-type; the other panels represent staurosporine (STS)-altered genes in wild-type and Δ*czt-1*, respectively. (B) Comparison between the percentage of upregulated (white) and downregulated (black) genes. (C and D) Venn diagrams showing the number of STS-induced (C) and STS-repressed (D) genes specifically in the wild-type or in the Δ*czt-1* mutant. FunCat functional enrichment analysis (Ruepp *et al.* 2004) for each set of genes is indicated (a, no highly enriched category was found).

High-throughput RNA-seq offers a far more accurate measurement of gene expression than DNA microarrays (Wang *et al.* 2009) and was used to analyze the patterns of gene expression in staurosporine-treated and control-treated wild-type cells. One hour of treatment with 20 μM staurosporine was sufficient to elicit a dynamic transcriptional response, with 28.1% of the genes having their expression significantly altered (Figure 5A, middle panel). From these, approximately half are induced (989 genes) and approximately half are repressed (1038 genes; Figure 5B, middle panel). The full expression data are presented in File S1. The results validate our previous microarray approach (Fernandes *et al.* 2011), although much more altered genes were found.

To understand the biological processes represented by these genes, we looked for functional enrichment among induced and repressed genes using FunCat. Although several genes are not cataloged by this database, it was possible to find a few enriched categories (Table 2 and File S2). On the upregulation side, there is a particular enrichment of “vesicular transport,” “modification with sugar residues,” and “fatty acid metabolism.” On the downregulation side, there is a very strong enrichment of some categories, namely those related to “ribosome biogenesis,” “rRNA processing,” “translation,” and “RNA binding.” It seems that the protein synthesis machinery is critically impaired by staurosporine. Genes encoding mitochondrion-related functions are also enriched in the staurosporine-repressed set of genes: “anaerobic respiration;” “aerobic respiration;” “electron transport;” “energy generation;” and “mitochondrial transport.” The downregulation of genes comprising the “respiration” category indicates that the treatment with staurosporine may cause a metabolic shift in the cells.

**Table 2:** Functional category enrichment analysis (using FunCat) of genes induced and repressed by staurosporine in the wild-type strain

**ID**	**Category**	**P-value**
Induced Genes		
01.05.02.07	Sugar, glucoside, polyol, and carboxylate catabolism	3.25E-05
01.06.05	Fatty acid metabolism	1.63E-05
14.07.02	Modification with sugar residues (*e.g.*, glycosylation, deglycosylation)	9.28E-06
16.17.07	Magnesium binding	8.28E-05
20.09.07	Vesicular transport (Golgi network, etc)	1.07E-06
Repressed Genes		
01.03	Nucleotide/nucleoside/nucleobase metabolism	1.89E-05
01.05.13	Transfer of activated C-groups	7.38E-08
02.11	Electron transport and membrane-associated energy conservation	1.60E-05
02.13.01	Anaerobic respiration	7.10E-05
02.13.03	Aerobic respiration	1.91E-09
02.45.15	Energy generation (*e.g.*, ATP synthase)	4.03E-06
11.02.01	rRNA synthesis	8.37E-14
11.02.02	tRNA synthesis	9.50E-06
11.04.01	rRNA processing	9.03E-46
11.06.01	rRNA modification	1.22E-07
12.01	Ribosome biogenesis	1.51E-137
12.01.01	Ribosomal proteins	8.44E-105
12.04	Translation	1.50E-65
12.07	Translational control	1.31E-08
16.01	Protein binding	1.38E-23
16.03.03	RNA binding	1.70E-32
20.01.15	Electron transport	1.56E-05
20.09.04	Mitochondrial transport	1.97E-12
42.16	Mitochondrion	8.43E-08

We also analyzed transcriptional patterns of the *czt-1* deletion strain (File S1). Considering the basal expression, 8.5% of the genes are altered in the Δ*czt-1* strain, corresponding to 605 genes (Figure 5A, left panel). From these, 58.5% were induced whereas 41.5% were repressed (Figure 5B, left panel). Enrichment analysis (File S2) shows, for instance, that because the mutant shows induction of these genes, CZT-1 negatively regulates “phosphate metabolism” and “cAMP/cGMP mediated signal transduction” (Table 3). However, it seems that CZT-1 is controlling positively the expression of genes involved in “respiration” and “alcohol fermentation” (Table 3).

**Table 3:** Functional category enrichment analysis (using FunCat) of genes induced and repressed basally in the Δ*czt-1* strain

**ID**	**Category**	**P-value**
Induced Genes		
01.04	Phosphate metabolism	7.37E-05
11.02.03.04	Transcriptional control	8.31E-06
30.01.09.07	cAMP/cGMP-mediated signal transduction	3.70E-05
36.25.01.13	Olfaction	4.57E-05
Repressed Genes		
02.13	Respiration	3.78E-05
02.16.01	Alcohol fermentation	1.72E-05

The response provoked by staurosporine in the Δ*czt-1* mutant is more active than in wild-type (File S1 and Figure S4), with 45.5% of the genes depicting altered expression (Figure 5A, right panel). Approximately half of these genes are induced and half are repressed (Figure 5B, right panel). The transcriptional response to staurosporine is partially independent of CZT-1 (Figure 5, C and D, note the number of shared genes). However, it is also true that CZT-1 is a major regulator of the genetic response to staurosporine, given that more than 1000 genes are only induced or repressed in the mutant strain and not in the wild-type (Figure 5, C and D).

A FunCat enrichment analysis for each group is represented in the Venn diagrams in Figure 5, C and D (and in File S3) for induced and repressed genes, respectively. On treatment with staurosporine, only wild-type cells are able to induce genes involved in several ER-related functions, such as “protein folding and stabilization,” “modification with sugar residues (*e.g.*, glycosylation, deglycosylation),” “ER to Golgi transport,” and “cellular export and secretion” (Figure 5C), suggesting an ER-mediated stress response that is not induced in Δ*czt-1*. Actually, the “protein folding and stabilization” category is significantly repressed in the mutant (Figure 5D). This suggests that CZT-1 affects ER processes. Signal transduction seems to be impaired in the deletion strain considering its inability to induce genes in the “second messenger mediated signal transduction” category, in opposition to the wild-type (Figure 5C). Within this category, the wild-type enrichment analysis includes the “polyphosphoinositol mediated signal transduction” subcategory (P-value = 0.0016), which seems to be regulated by CZT-1. As described above, wild-type cells downregulate genes involved in the mitochondrial electron transport after treatment with staurosporine (Table 2). Interestingly, this is not the case in *czt-1* knockout cells (Figure 5D).

Although the Gene Ontology (GO) annotation of Neurospora genes is not yet completely developed, we wanted to validate the FunCat results with the available GO annotation. We used Δ*czt-1*-treated cells as the test set and wild-type-treated cells as the reference set. This allows the estimation of over-representation and under-representation of GO terms in the mutant using the wild-type as a reference. The results essentially validate the FunCat analysis, with some extra information (File S4). There is an under-representation of “endoplasmic reticulum membrane” in the set of induced genes in the Δ*czt-1* strain. In the repressed set of genes, the GO analysis shows an under-representation of “cytochrome-c oxidase activity” and “ATP synthase activity,” indicating a role for CZT-1 in the regulation of genes related to complex IV and complex V of the electron transport chain. Interestingly, the “response to light stimulus” GO term is over-represented in the set of induced genes and under-represented in the set of repressed genes, pointing to a possible new role of CZT-1 in the light response pathway.

**Figure 6:**
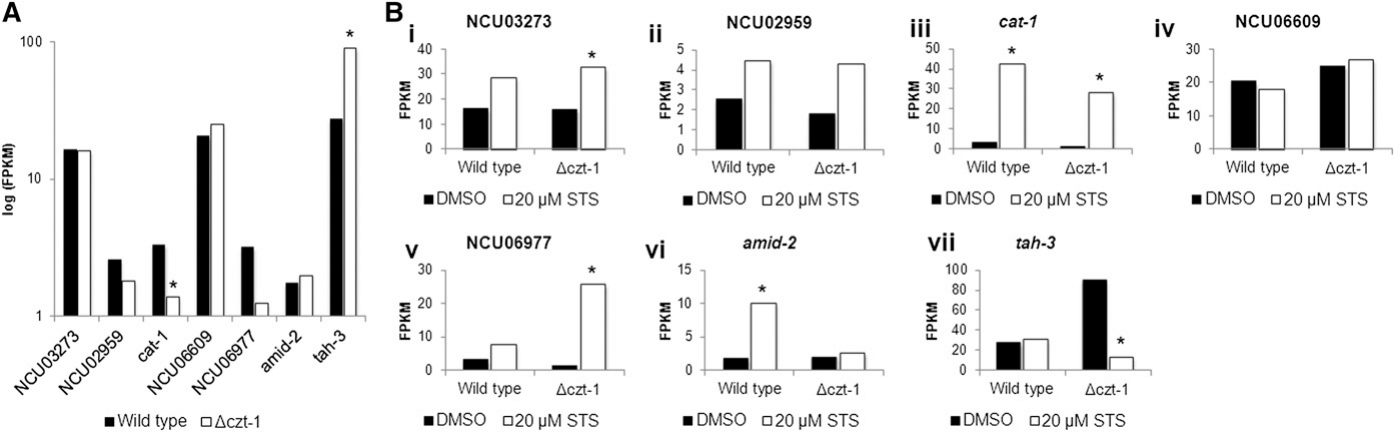
RNA-seq data support the involvement of genes identified by the GWAS in staurosporine-induced cell death. (A and B) The expression of NCU03273, NCU02959, *cat-1*, NCU06609, NCU06977, *amid-2*, and *tah-3*, indicated as FPKMs, was compared between wild-type and Δ*czt-1* strains in control samples (A) or between staurosporine (STS)-treated and DMSO-treated samples (B). *P-value <0.05.

We examined whether there were differences in expression in wild-type *vs.* Δ*czt-1* strain for the genes identified by the GWAS (Figure 4A). At basal conditions, Δ*czt-1* shows significant downregulation of *cat-1* and upregulation of *tah-3* when compared with wild-type (Figure 6A). On treatment with staurosporine, *amid-2* is induced by the drug in the wild-type but not in the mutant; NCU06977 is induced by the drug in Δ*czt-1* but not in the wild-type; *tah-3* is repressed by the drug in the mutant but not in the wild-type; and *cat-1* is induced by staurosporine in both strains, although more extensively in Δ*czt-1* (Figure 6B). The alteration in the expression of *cat-1* is in agreement with the fact that staurosporine induces ROS formation, which is increased in Δ*czt-1*. We also studied expression alterations of the *amid-2* homologues, namely *amid* and the apoptosis-inducing factor (*aif*): in the presence of staurosporine, *amid* expression is repressed only in the mutant, whereas aif was induced in both strains (File S1). The staurosporine-provoked expression profile for all these genes corroborate their role in cell death and expression differences observed in Δ*czt-1* suggest that they are under the control of the transcription factor.

### CZT-1 regulates the expression of multiple ABC transporters

**Figure 7:**
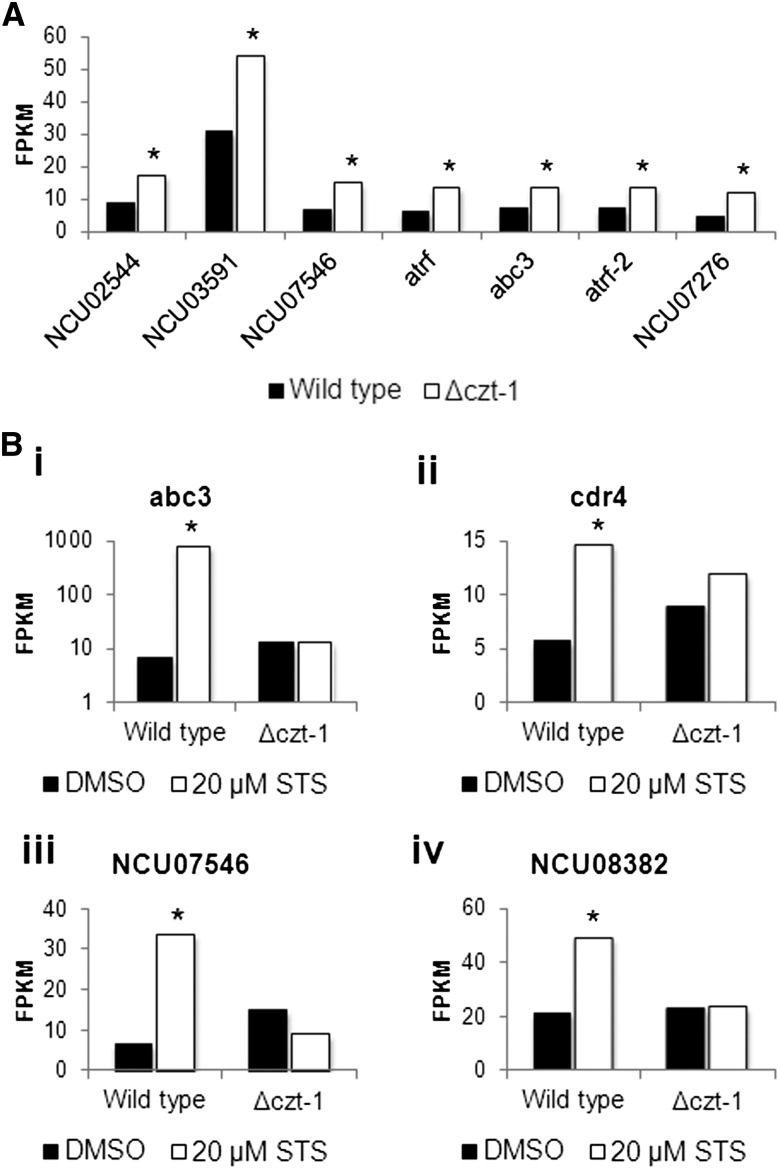
CZT-1 controls genes encoding ABC transporters. (A) The levels of expression (in FPKMs) of the indicated ABC transporter–encoding genes were compared between wild-type and Δ*czt-1* strains under basal conditions. (B) Expression of *abc3*, *cdr4*, NCU07546, and NCU08382, indicated as FPKMs, was compared between wild-type and Δ*czt-1* strains treated with staurosporine or DMSO. *P-value <0.05.

Genes encoding members of the ABC (ATP-binding cassette)-transporter family are induced in response to drug treatments and confer resistance to numerous compounds in a process designated multidrug drug resistance or pleotropic drug resistance (Gulshan and Moye-Rowley 2007). We compiled a list of genes encoding ABC transporters from the FunCat database and checked their expression in the RNA-seq dataset. Under basal conditions, there is an upregulation of some of these genes in Δ*czt-1* cells (Figure 7A and File S5), including NCU02544, NCU03591, NCU07546, NCU08056/*atrf*, NCU10009/*atrf-2*, NCU07276, and NCU09975/*abc3*. The latter gene plays a crucial role during the response of *N. crassa* to staurosporine since we observed that the cells pump the drug out to the extracellular medium through ABC3, which seems to be influenced by CZT-1 (Fernandes *et al.* 2011). The staurosporine-stimulated induction of *abc3* (NCU09975) in the wild-type but not in Δ*czt-1* cells was confirmed by our RNA-seq data (Figure 7B, i). Interestingly, *czt-1* and *abc3* are adjacent in the genome, indicating a functional link between neighboring genes. On treatment of wild-type cells with staurosporine, not only *abc3* but also other ABC transporter genes are induced. These include NCU05591/*cdr4* (Figure 7B, ii), NCU07546 (Figure 7B, iii), and NCU08382 (Figure 7B, iv). CDR4 was shown to be involved in resistance to azoles in Neurospora (Zhang *et al.* 2012). Cells lacking CZT-1 are unable to induce these genes, indicating that the expression of several ABC transporters is CZT-1-dependent. Altogether, our data show that the novel transcription factor CZT-1 is an important regulator of cell death and controls the genetic response to staurosporine in *N. crassa*.

## Discussion

The protein encoded by NCU09974, now termed CZT-1, was not considered before in transcription factor–related works in *N. crassa*, likely because only a recent genome annotation added a few extra exons to the gene that include a Zn_2_/Cys_6_ DNA-binding domain. We show that CZT-1 is a crucial mediator of the fungal response to staurosporine in laboratory strains as well as in wild isolates of Neurospora. Transcriptional profiling of the Δ*czt-1* mutant suggests that CZT-1 controls the expression of several genes, affecting diverse processes and organelles, including the mitochondria and the ER. This regulatory role is in accordance with the transcription factor–related sequence features of CZT-1. The presence of a conserved Zn_2_/Cys_6_ DNA-binding domain at the N-terminal portion of CZT-1 positions this protein in the zinc cluster family of transcription factors, which is found exclusively in fungi, namely in the Ascomycota phylum (MacPherson *et al.* 2006). CZT-1 homologs from BLAST searches correspond mostly to uncharacterized proteins in diverse fungi, including human pathogens such as *Aspergillus fumigatus* or crop pathogens like *Magnaporthe oryzae* or *Fusarium sp*. There is strong homology between CZT-1 and members of the Pezizomycotina, whereas homology is weaker for members of the Saccharomycotina and Taphrinomycotina. Furthermore, within the same species, it appears that there are two types of zinc cluster proteins, some with strong homology to CZT-1 and others with weak homology. Specific CZT-1 sequence features suggest that it may belong to a novel divergent subfamily of zinc cluster transcription factors (Figure S5). Thus, we consider that our data on the functions of CZT-1 have a broad interest and can be the basis of future studies of the role of this uncharacterized group of proteins that may have an important role in antifungal responses and drug resistance in clinical or economic relevant fungi.

A large number of zinc cluster transcription factors regulate drug resistance, consistent with our observations on the role of CZT-1. Multidrug resistance is highly conserved and commonly activated by microbes or cancer cells on exposure to cellular insults. It is the consequence of the extrusion of drugs by cells overexpressing pumps of the ABC transporter family or the major facilitator superfamily (MacPherson *et al.* 2006). In *C. albicans*, the zinc cluster transcription factor Tac1 controls the expression of the ABC transporter genes *CDR1* and *CDR2*, which mediate resistance to azole drugs (Coste *et al.* 2004). In *Saccharomyces cerevisiae*, some of these regulators, including Pdr1, Pdr3, Yrr1, and Stb5, interact as homodimers and heterodimers to accomplish their regulatory functions (Akache *et al.* 2004; Mamnun *et al.* 2002).

Stb5 is an oxidative stress–induced transcription factor that regulates multidrug resistance (Akache and Turcotte 2002) as well as the expression of genes in the pentose phosphate pathway affecting NADPH production (Larochelle *et al.* 2006). This may parallel to some extent the role of CZT-1 in Neurospora, since the lack of the protein results in a higher accumulation of ROS on insult with staurosporine. The deletion of *czt-1* results in altered expression of several components of the oxidative stress detoxification machinery, both basally and after addition of the drug (File S6). In addition, CZT-1 is necessary for the induction of ABC3, and the ABC3 homolog in *Magnaporthe grisea* is important for cell survival during oxidative stress (Sun *et al.* 2006). RNA-seq analyses also show that diverse genes encoding molecules intervening in intracellular Ca^2+^ handling are altered in *czt-1* mutant cells (File S7).

We observed that the lack of *czt-1* seems to be particularly important for the response to staurosporine compared to other tested stimuli (the lack of *czt-1* results in a small increase in sensitivity to phytosphingosine and cinnamic acid and resistance to amphotericin B). However, we tested the susceptibility of Δ*czt-1* to a limited number of drugs; therefore, the possibility that CZT-1 is essential to provide resistance to other stresses is an open subject. The expression of *czt-1* is induced by a number of compounds and CZT-1 controls different ABC transporters, which are mediators of multidrug resistance. Thus, it is tempting to speculate that the observed variation in resistance to staurosporine for the wild isolates of the fungus may have implications in the response to certain conditions present in their natural habitats. We looked for biogeographic correlations between the expression of *czt-1* and the collection place of the wild strains (data not shown) and, although we did not achieve strong statistical significance, but considering the relatively small size of the population, some interesting associations were found. This includes a tendency toward high expression of *czt-1* and, consequently, resistance to staurosporine for strains collected in Iowa, Louisiana, or Elizabeth, Louisiana, east and north of Lake Charles, respectively.

Natural variation to oxidative stress and drug resistance has been shown in other fungi. In *S. cerevisiae*, clinical and soil isolates from Pennsylvania (USA) tolerate ROS accumulation better than human-associated brewery and vineyard strains, soil isolates from North Carolina, or fruit populations, indicating that response to oxidative stress is an adaptive feature (Diezmann and Dietrich 2009). Recently, a GWAS associated a quantitative trait nucleotide in *RDS2* with increased survival on oxidative stress in a clinical isolate of *S. cerevisiae vs.* a laboratory reference strain (Diezmann and Dietrich 2011), and *RDS2* encodes a zinc cluster transcription factor (Akache and Turcotte 2002). In the plant pathogen *Botrytis cinerea*, three natural populations of field isolates from vineyards in France and Germany exhibiting different mechanisms of fungicide resistance were identified (Kretschmer *et al.* 2009). In one of these populations, all strains had mutations in a zinc cluster transcription factor–encoding gene, *mrr1*, causing overexpression of the ABC transporter AtrB (Kretschmer *et al.* 2009). Resistance to sterol demethylation inhibitors and strobilurins in field isolates of *Penicillium digitatum* and *Mycosphaerella graminicola*, respectively, was also described (Nakaune *et al.* 1998; Roohparvar *et al.* 2008). Our work using a subtropical wild population of *N. crassa* (Ellison *et al.* 2011; Palma-Guerrero *et al.* 2013) further supports that natural variation to drug tolerance occurs and seems to be mediated by zinc cluster transcription factors. A GWAS using this population pointed to putative loci involved in the process, including the oxidative stress–related and cell death–related proteins CAT-1 and AMID-2, respectively.

We showed that expression of different ABC transporters is CZT-1-dependent. This is likely to be related to the drug resistance regulatory role of the transcription factor. Because the inactivation of *czt-1* leads to deficiency in several drug efflux pumps, it may be a good target to overcome drug resistance. This suggestion makes it relevant to characterize, in future studies, the role of CZT-1 and its homologs in pathogenic organisms.

## Supplementary Material

Supporting Information
