# Supplementary material for: CZT-1 Is a Novel Transcription Factor Controlling Cell Death and Natural Drug Resistance in Neurospora crassa
Source: G3 (Bethesda). 2014 Apr 8;4(6):1091–102. doi: 10.1534/g3.114.011312 (PMC4065252; doi:10.1534/g3.114.011312)
Supplement: Supporting Information [file supp_4_6_1091__index.html]

CZT-1 Is a Novel Transcription Factor Controlling Cell Death and Natural Drug Resistance in Neurospora crassa — Supporting Information 

# CZT-1 Is a Novel Transcription Factor Controlling Cell Death and Natural Drug Resistance in *Neurospora crassa*

## Supporting Information for Gonçalves *et al.*, 2014

**Files in this Data Supplement:**

- Supporting Information - Figures S1-S5 and Files S1-S7 (PDF, 618 KB)
- Figure S1 - Sensitivity profile of the wild type and ΔNCU09974 strains on GFS medium supplemented with phytosphingosine (PHS) (A), cinnamic acid (B) and amphotericin B (AmphoB) (C). (PDF, 236 KB)
- Figure S2 - Expression levels of *czt-1*, expressed as RPKM, in wild strains of *Neurospora* collected in the Louisiana state (USA). (PDF, 154 KB)
- Figure S3 - Box plots showing the relationship between the two found nucleotides for each SNP identified by the GWAS and the expression of the respective gene. (PDF, 161 KB)
- Figure S4 - Volcano plots illustrate the amplification of the response to staurosporine in Δ*czt-1* versus the wild type strain. (PDF, 321 KB)
- Figure S5 - A maximum likelihood phylogenetic tree was built using the Dayhoff method on a ClustalW2 multiple alignment. (PDF, 232 KB)
- File S1 - Complete RNA-seq dataset. (.xlsx, 2 MB)
- File S2 - Complete functional enrichment analysis, using FunCat, of genes whose expression was altered by staurosporine in wild type cells and genes whose expression is basally affected by deletion of *czt-1*. (.xlsx, 333 KB)
- File S3 - Complete functional enrichment analysis, using FunCat, of genes whose expression was specifically induced or repressed only in wild type, only in Δ*czt-1* or in both strains. (.xlsx, 321 KB)
- File S4 - Blast2GO analysis of functional enrichment of staurosporine-induced or -repressed genes using Δ*czt-1*-treated cells as the test set and wild type-treated cells as the reference set. (.xlsx, 39 KB)
- File S5 - Expression levels comparison between wild type and Δ*czt-1* cells for genes encoding ABC transporters. (.xlsx, 60 KB)
- File S6 - Expression levels comparison between wild type and Δ*czt-1* cells for genes involved in oxidative stress. (.xlsx, 64 KB)
- File S7 - Expression levels comparison between wild type and Δ*czt-1* cells for genes involved in Ca2+ handling. (.xlsx, 58 KB)
