# Supplementary material for: CZT-1 Is a Novel Transcription Factor Controlling Cell Death and Natural Drug Resistance in Neurospora crassa
Source: G3 (Bethesda). 2014 Apr 8;4(6):1091–102. doi: 10.1534/g3.114.011312 (PMC4065252; doi:10.1534/g3.114.011312)
Supplement: Supporting Information [file supp_g3.114.011312_011312SI.pdf]

G3 · Genes | Genomes | Genetics

CZT-1 is a novel transcription factor controlling cell death and natural drug resistance in *Neurospora crassa*

A. Pedro Gonçalves * §, Charles Hall † [1], David J. Kowbel †, N. Louise Glass †, Arnaldo Videira * §

* IBMC - Instituto de Biologia Molecular e Celular - Universidade do Porto, Rua do Campo Alegre 823, 4150-180 Porto, Portugal
§ ICBAS - Instituto de Ciências Biomédicas de Abel Salazar, Universidade do Porto, Rua de Jorge Viterbo Ferreira 228, 4050-313 Porto, Portugal
† Plant and Microbial Biology Department, The University of California, Berkeley, 341 Koshland Hall, 94720, Berkeley, CA, USA

[1] Current address: Dupont Industrial Biosciences, 925 Page Mill Road, 94304, Palo Alto, CA, USA

RNA-sequencing data from this study have been submitted to the NCBI Gene Expression Omnibus (GEO) (http://www.ncbi.nlm.nih.gov/geo); series record: GSE52153.

Corresponding authors:
António Pedro Gonçalves
ICBAS - Instituto de Ciências Biomédicas de Abel Salazar, Universidade do Porto
Genética Molecular - Edifício 1, Piso 5
Rua de Jorge Viterbo Ferreira 228,
4050-313 Porto, Portugal
apgoncalves@ibmc.up.pt

Arnaldo Videira
ICBAS - Instituto de Ciências Biomédicas de Abel Salazar, Universidade do Porto
Genética Molecular - Edifício 1, Piso 5
Rua de Jorge Viterbo Ferreira 228,
4050-313 Porto, Portugal
avideira@ibmc.up.pt

DOI: 10.1534/g3.114.011312

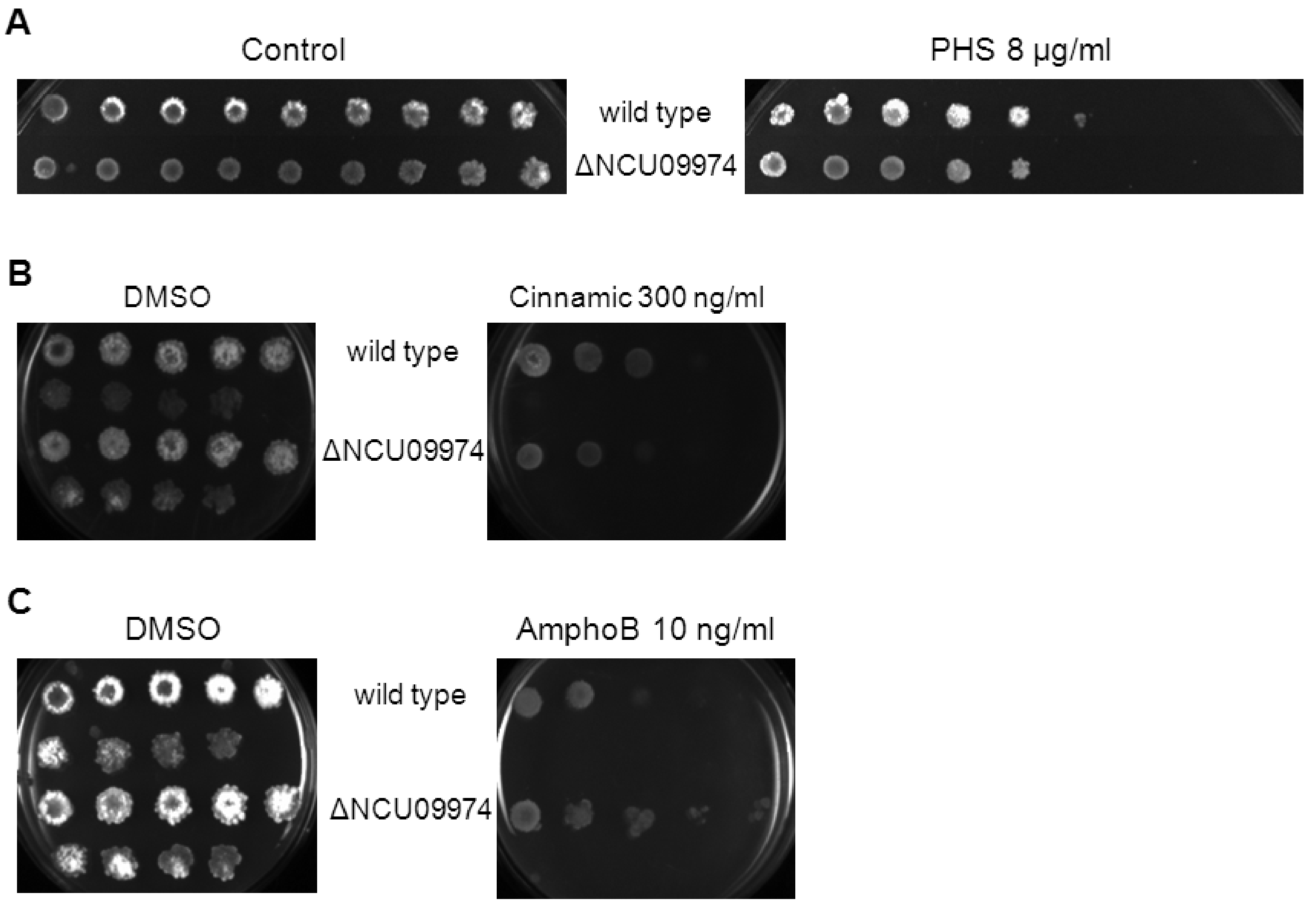

Figure S1 Sensitivity profile of the wild type and ΔNCU09974 strains on GFS medium supplemented with phytosphingosine (PHS) (A), cinnamic acid (B) and amphotericin B (AmphoB) (C).

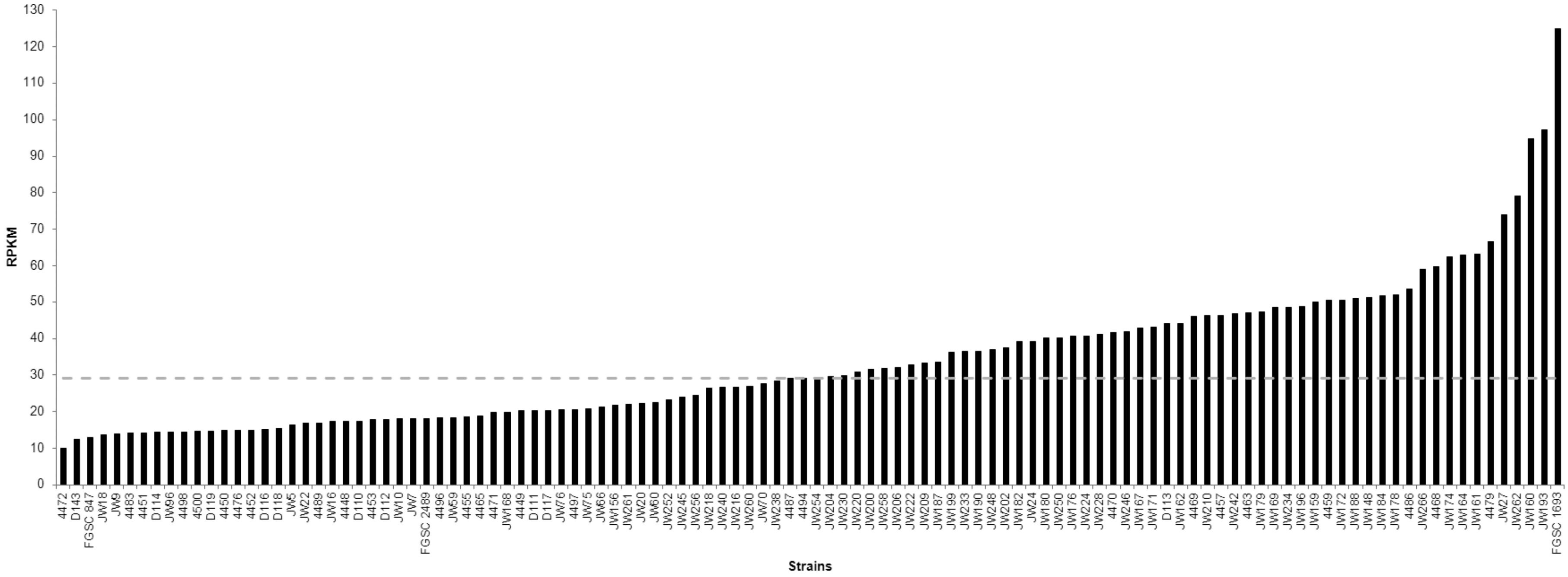

Figure S2 Expression levels of *czt-1*, expressed as RPKM, in wild strains of *Neurospora* collected in the Louisiana state (USA). The wild type laboratory strain FGSC 2489 is shown for reference. The broken line represents the median RPKM.

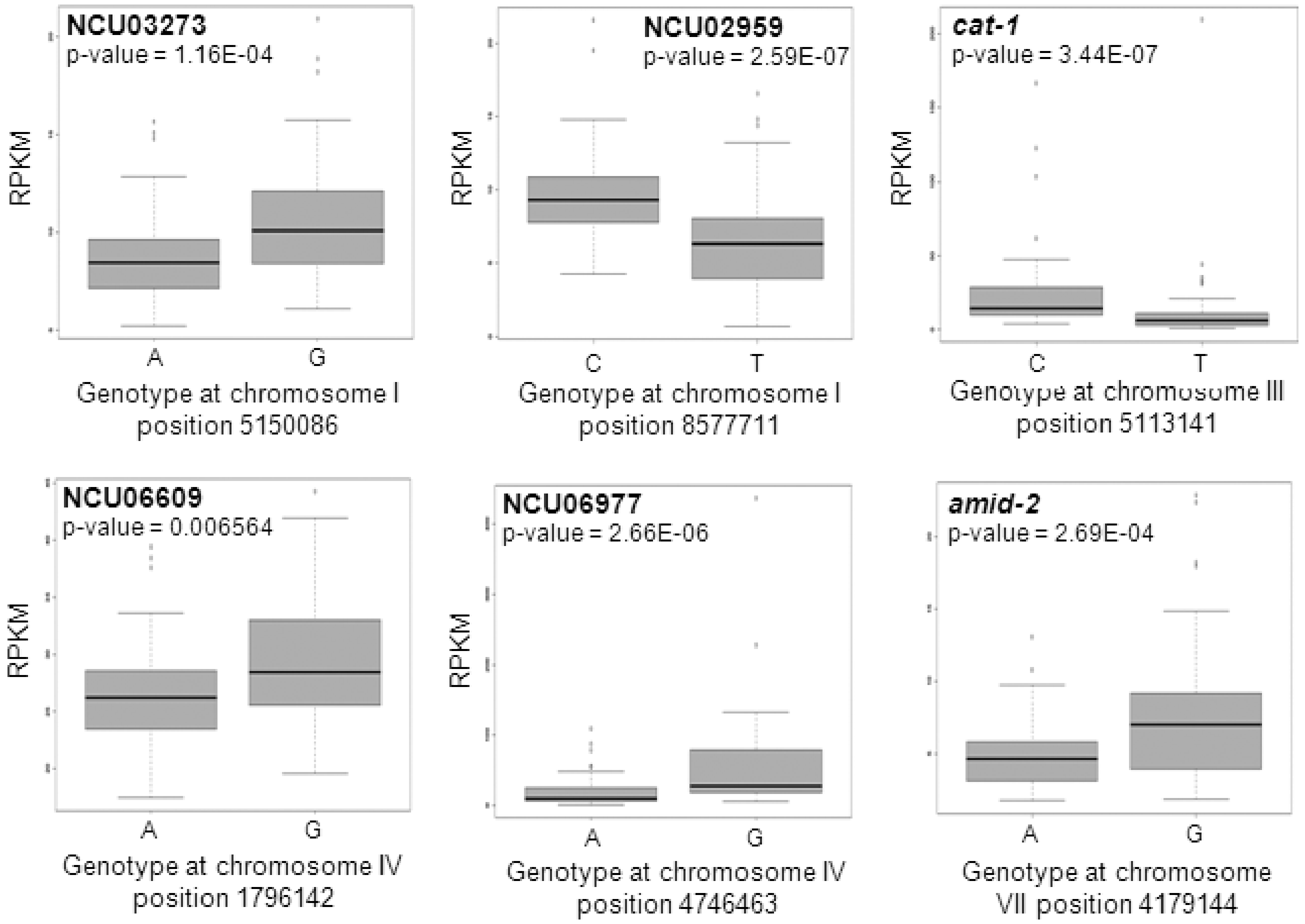

Figure S3 Box plots showing the relationship between the two found nucleotides for each SNP identified by the GWAS and the expression of the respective gene.

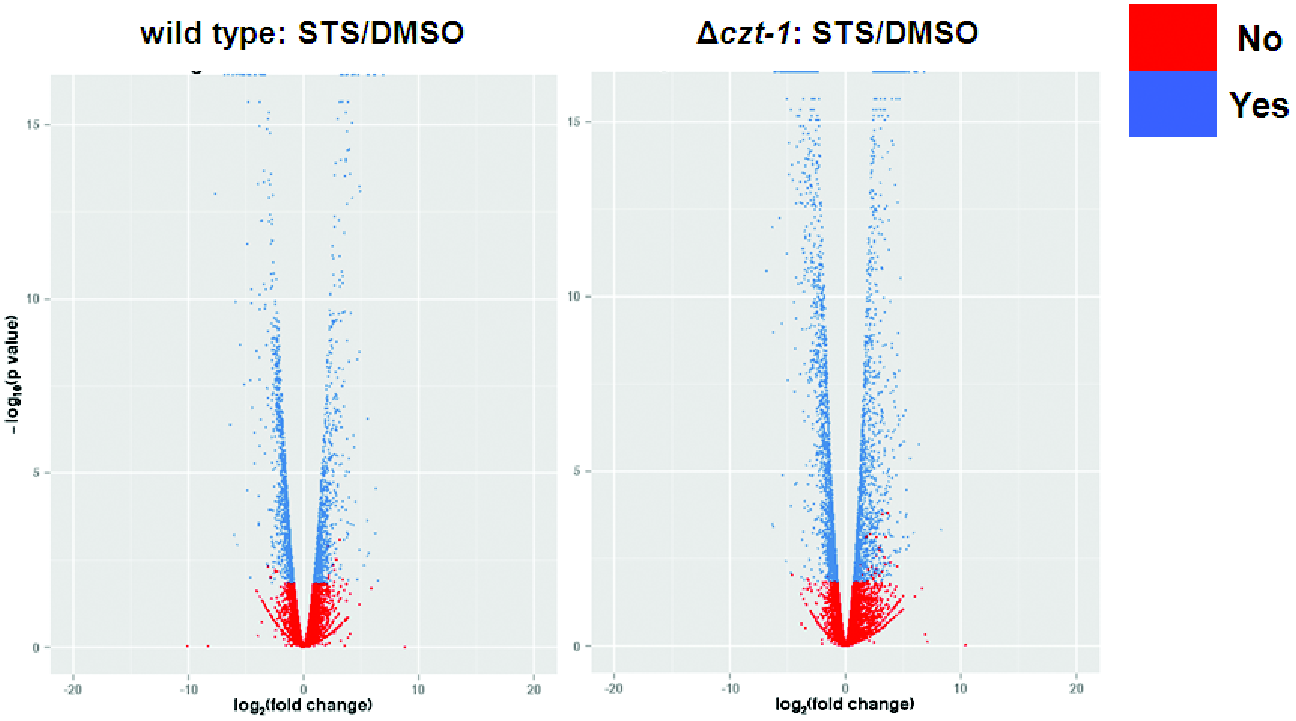

Figure S4 Volcano plots illustrate the amplification of the response to staurosporine in Δ*czt-1* *versus* the wild type strain.

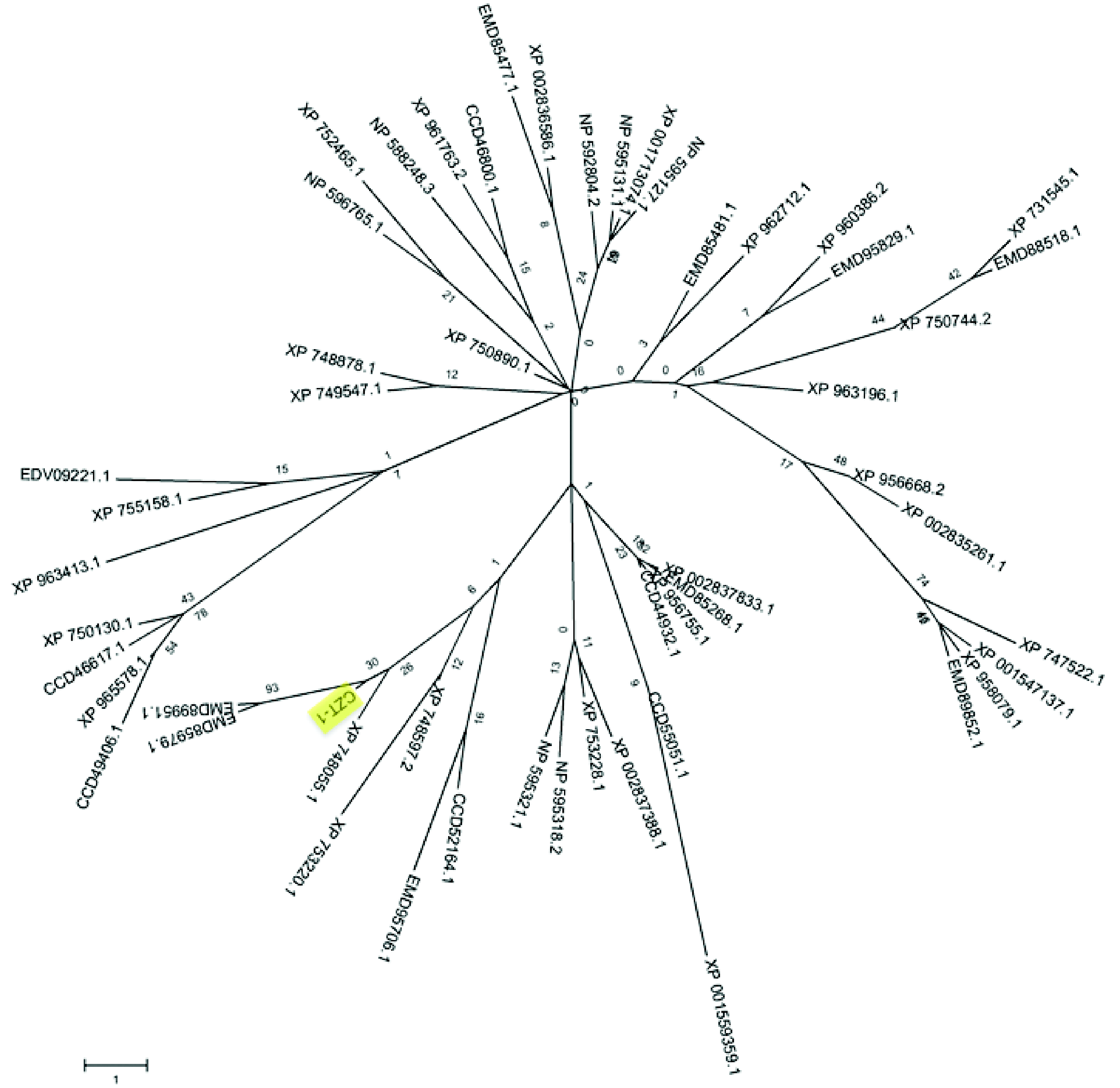

Figure S5 A maximum likelihood phylogenetic tree was built using the Dayhoff method on a ClustalW2 multiple alignment. The bootstrap test (1000 replications) was applied. Different classes of Ascomycota are represented (one species per class): Sordariomycetes/*Neurospora crassa* (accession numbers: XP_958092.1/CZT-1, XP_956755.1, XP_963196.1, XP_961763.2, XP_958079.1, XP_956668.2, XP_963413.1, XP_960386.2, XP_965578.1, XP_962712.1), Eurotiomycetes/*Aspergillus fumigatus* (XP_748055.1, XP_748597.2, XP_753220.1, XP_753228.1, XP_755158.1, XP_750744.2, XP_731545.1, XP_747522.1, XP_749547.1, XP_750130.1, XP_748878.1, XP_752465.1, XP_750890.1), Dothideomycetes/*Bipolaris maydis* (EMD85979.1, EMD89951.1, EMD85481.1, EMD85477.1, EMD85268.1, EMD89852.1, EMD88518.1, EMD95829.1, EMD95706.1), Pezizomycetes/*Tuber melanosporum* (XP_002837388.1, XP_002837833.1, XP_002836586.1, XP_002835261.1), Leotiomycetes/*Botrytis cinerea* (CCD52164.1, CCD44932.1, CCD46036.1, CCD55051.1, CCD49406.1, CCD46800.1, CCD43955.1, CCD46617.1), Saccharomycetes/*Saccharomyces cerevisiae* (EDV09221.1) and Schizosaccharomycetes/*Schizosaccharomyces pombe* (XP_001713074.1, NP_592804.2, NP_595127.1, NP_596765.1, NP_588248.3, NP_595318.2, NP_595131.1, NP_595321.1). CZT-1 is highlighted by a yellow-shaded box.

Files S1-S7

Available for download as Excel files at http://www.g3journal.org/lookup/suppl/doi:10.1534/g3.114.011312/-/DC1

File S1 Complete RNA-seq dataset.

File S2 Complete functional enrichment analysis, using FunCat, of genes whose expression was altered by staurosporine in wild type cells and genes whose expression is basally affected by deletion of *czt-1*.

File S3 Complete functional enrichment analysis, using FunCat, of genes whose expression was specifically induced or repressed only in wild type, only in Δ*czt-1* or in both strains.

File S4 Blast2GO analysis of functional enrichment of staurosporine-induced or -repressed genes using Δ*czt-1*-treated cells as the test set and wild type-treated cells as the reference set.

File S5 Expression levels comparison between wild type and Δ*czt-1* cells for genes encoding ABC transporters.

File S6 Expression levels comparison between wild type and Δ*czt-1* cells for genes involved in oxidative stress.

File S7 Expression levels comparison between wild type and Δ*czt-1* cells for genes involved in Ca^{2+} handling.
