# Supplementary material for: CZT-1 Is a Novel Transcription Factor Controlling Cell Death and Natural Drug Resistance in Neurospora crassa
Source: G3 (Bethesda). 2014 Apr 8;4(6):1091–102. doi: 10.1534/g3.114.011312 (PMC4065252; doi:10.1534/g3.114.011312)
Supplement: Supporting Information [file supp_g3.114.011312_FigureS1.pdf]

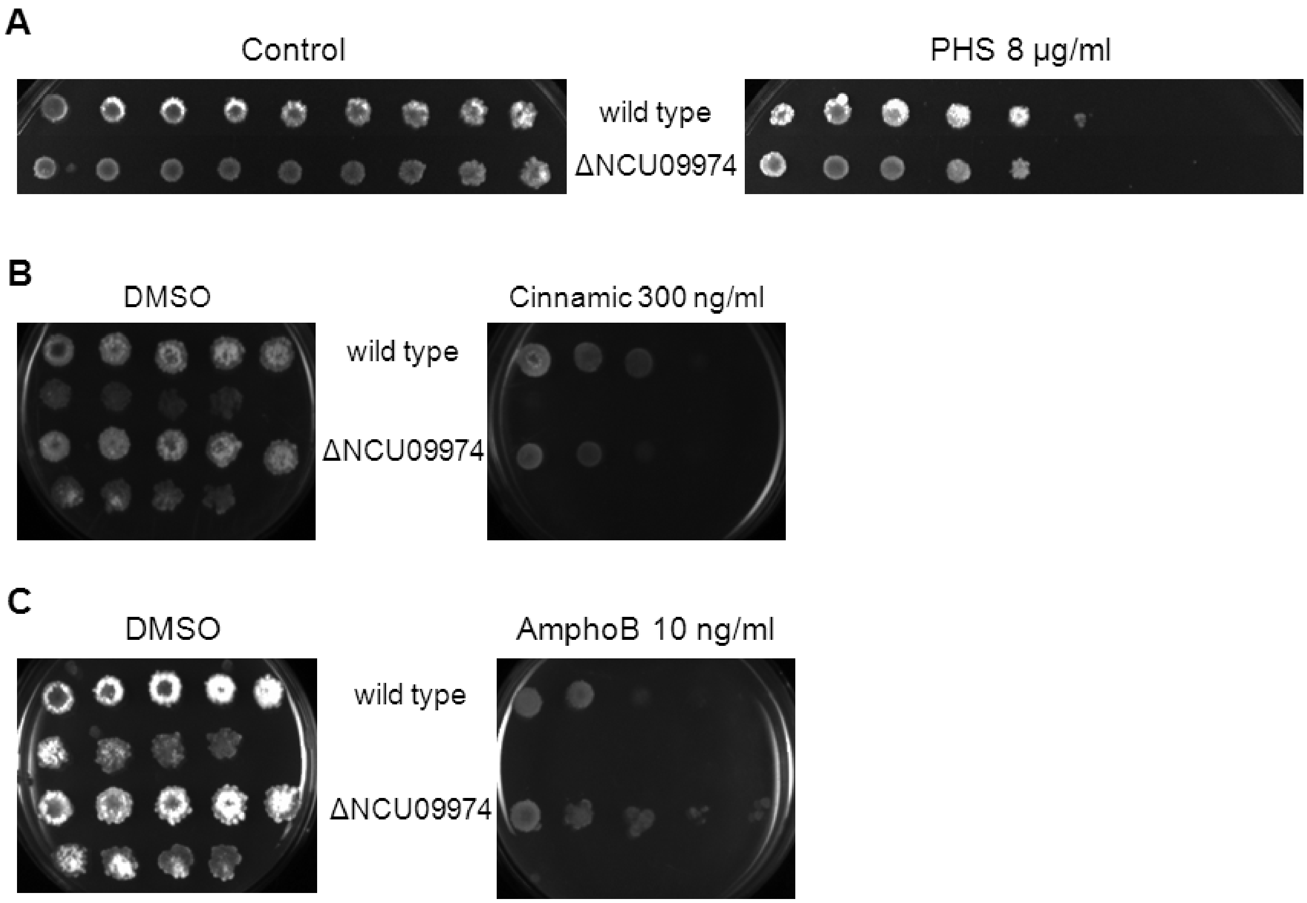

Figure S1 Sensitivity profile of the wild type and ΔNCU09974 strains on GFS medium supplemented with phytosphingosine (PHS) (A), cinnamic acid (B) and amphotericin B (AmphoB) (C).
