# Supplementary material for: CZT-1 Is a Novel Transcription Factor Controlling Cell Death and Natural Drug Resistance in Neurospora crassa
Source: G3 (Bethesda). 2014 Apr 8;4(6):1091–102. doi: 10.1534/g3.114.011312 (PMC4065252; doi:10.1534/g3.114.011312)
Supplement: Supporting Information [file supp_g3.114.011312_FigureS2.pdf]

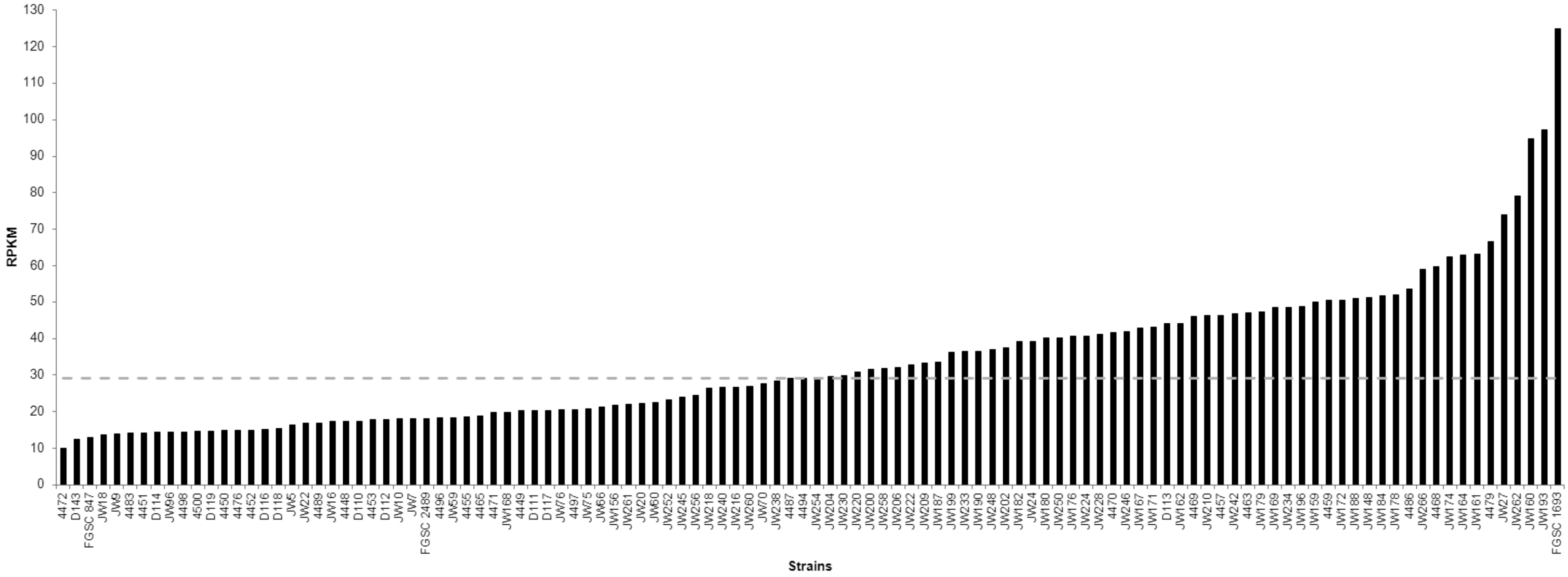

Figure S2 Expression levels of *czt-1*, expressed as RPKM, in wild strains of *Neurospora* collected in the Louisiana state (USA). The wild type laboratory strain FGSC 2489 is shown for reference. The broken line represents the median RPKM.
