# Supplementary material for: CZT-1 Is a Novel Transcription Factor Controlling Cell Death and Natural Drug Resistance in Neurospora crassa
Source: G3 (Bethesda). 2014 Apr 8;4(6):1091–102. doi: 10.1534/g3.114.011312 (PMC4065252; doi:10.1534/g3.114.011312)
Supplement: Supporting Information [file supp_g3.114.011312_FigureS5.pdf]

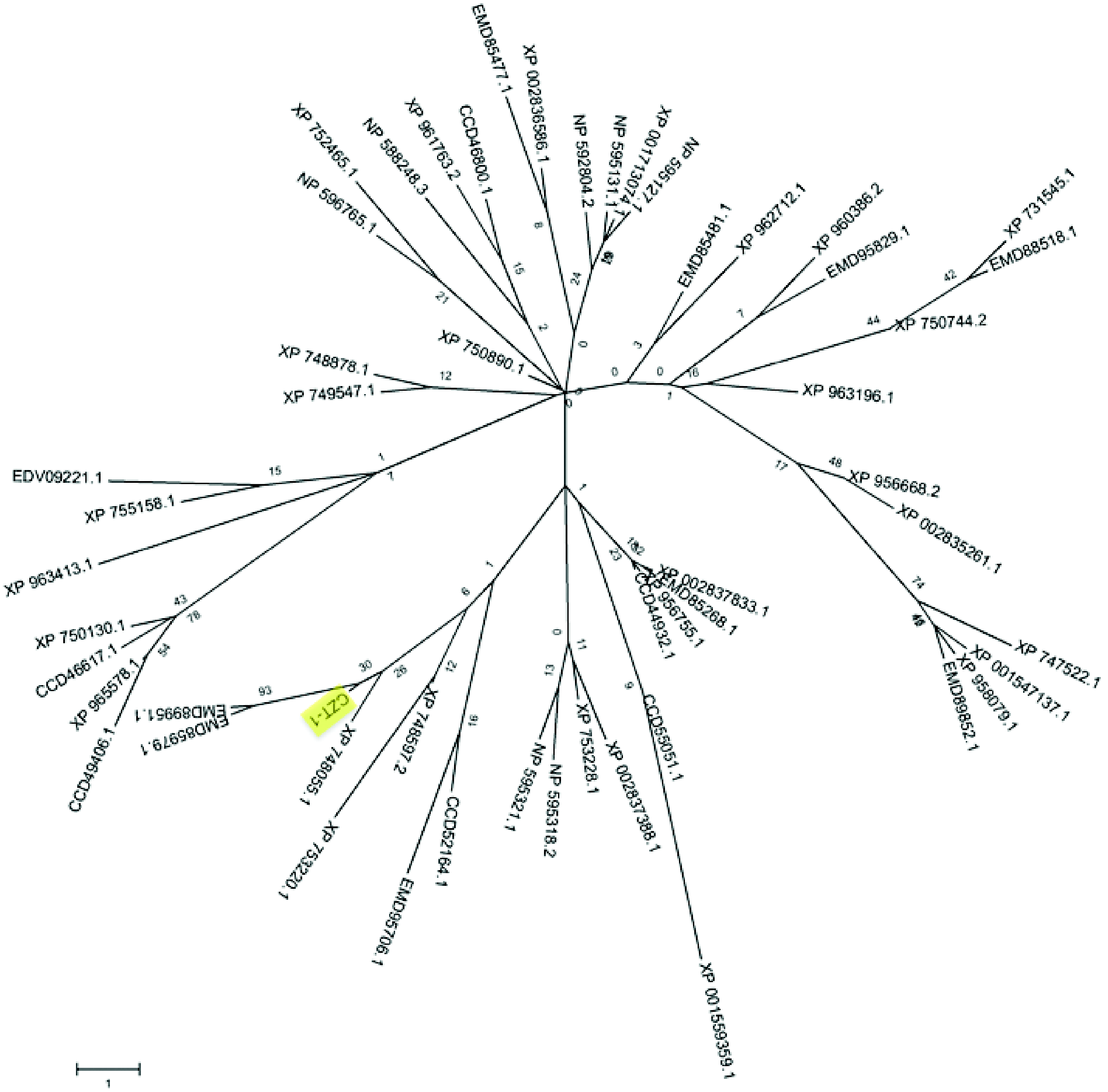

Figure S5 A maximum likelihood phylogenetic tree was built using the Dayhoff method on a ClustalW2 multiple alignment. The bootstrap test (1000 replications) was applied. Different classes of Ascomycota are represented (one species per class): Sordariomycetes/*Neurospora crassa* (accession numbers: XP_958092.1/CZT-1, XP_956755.1, XP_963196.1, XP_961763.2, XP_958079.1, XP_956668.2, XP_963413.1, XP_960386.2, XP_965578.1, XP_962712.1), Eurotiomycetes/*Aspergillus fumigatus* (XP_748055.1, XP_748597.2, XP_753220.1, XP_753228.1, XP_755158.1, XP_750744.2, XP_731545.1, XP_747522.1, XP_749547.1, XP_750130.1, XP_748878.1, XP_752465.1, XP_750890.1), Dothideomycetes/*Bipolaris maydis* (EMD85979.1, EMD89951.1, EMD85481.1, EMD85477.1, EMD85268.1, EMD89852.1, EMD88518.1, EMD95829.1, EMD95706.1), Pezizomycetes/*Tuber melanosporum* (XP_002837388.1, XP_002837833.1, XP_002836586.1, XP_002835261.1), Leotiomycetes/*Botrytis cinerea* (CCD52164.1, CCD44932.1, CCD46036.1, CCD55051.1, CCD49406.1, CCD46800.1, CCD43955.1, CCD46617.1), Saccharomycetes/*Saccharomyces cerevisiae* (EDV09221.1) and Schizosaccharomycetes/*Schizosaccharomyces pombe* (XP_001713074.1, NP_592804.2, NP_595127.1, NP_596765.1, NP_588248.3, NP_595318.2, NP_595131.1, NP_595321.1). CZT-1 is highlighted by a yellow-shaded box.
